# Benzodiazepines compromise the outcome of cancer immunotherapy

**DOI:** 10.1080/2162402X.2024.2413719

**Published:** 2024-10-07

**Authors:** Léa Montégut, Lisa Derosa, Meriem Messaoudene, Hui Chen, Flavia Lambertucci, Bertrand Routy, Laurence Zitvogel, Isabelle Martins, Guido Kroemer

**Affiliations:** a Centre de Recherche des Cordeliers, Equipe labellisée par la Ligue contre le cancer, Paris, France; b Metabolomics and Cell Biology Platforms, Gustave Roussy Institute, Villejuif, France; c INSERM U1015, Equipe Labellisée-Ligue Nationale contre le Cancer, Gustave Roussy Cancer Campus, Université Paris-Saclay, Villejuif, France; d Department of Biology, Center of Clinical Investigations in Biotherapies of Cancer (CICBT) BIOTHERIS, Villejuif, France; e Axe cancer, Centre de recherche du Centre Hospitalier de l’Université de Montréal (CRCHUM), Montréal, Canada; f Hemato-oncology Division, Centre Hospitalier de l’Université de Montréal (CHUM), Montréal, Canada; g Institut du Cancer Paris CARPEM, Department of Biology, Hôpital Européen Georges Pompidou, AP-HP, Paris, France

**Keywords:** Benzodiazepines, comedications, Immunotherapy, neuroendocrine factors, non-small cell lung cancer

## Abstract

Acyl CoA binding protein (ACBP, which is encoded by *diazepam binding inhibitor*, *DBI*) acts on the gamma-amino butyric acid (GABA) receptor type A via a specific binding site that is shared by diazepam and other benzodiazepines. Both ACBP/DBI and benzodiazepines act as positive allosteric modulators, hence increasing GABA effects on this receptor. Recently, we found that ACBP/DBI acts as an endogenous immunosuppressor, meaning that its antibody-mediated neutralization has immunostimulatory effects and enhances the efficacy of immunotherapy and chemoimmunotherapy in mouse models. Driven by these considerations, we investigated whether diazepam administration in mice would reverse the beneficial effects of ACBP/DBI neutralization on cancer chemoimmunotherapy. Indeed, diazepam abolished the therapeutic of anti-ACBP/DBI antibodies, supporting the idea that diazepam exerts immunosuppressive properties. Of note, treatment with benzodiazepines was associated with poor clinical responses to chemoimmunotherapy in patients with non-small cell lung cancer (NSCLC) as compared to individuals not receiving any psychotropic drugs. Medication with other psychotropic drugs than benzodiazepines did not compromise the outcome of chemoimmunotherapy, indicating that this immunosuppressive effect was benzodiazepine specific. We conclude that benzodiazepines may confer systemic immunosuppression. This hypothesis requires further epidemiological and clinical confirmation.

## Introduction

Oncoimmunological research is driven by the constant quest for new therapeutic targets. We recently discovered that antibody-mediated neutralization of acyl CoA binding protein (ACBP, which is encoded by *diazepam binding inhibitor*, *DBI*) can improve the outcome of immunotherapy or chemoimmunotherapy against several cancers including mouse models of breast carcinoma, cutaneous fibrosarcoma and non-small cell lung cancer.^1^ This discovery was driven by the considerations that (i) immunosurveillance is the most impactful determinant of the success of anticancer treatments,^2,3^ (ii) that aging and obesity are the most important risk factors of neoplastic diseases,^4,5^ that (iii) ACBP/DBI is a tissue hormone that increases with age and overweight/obesity,^6,7^ and (iv) that circulating ACBP/DBI is particularly elevated in still apparently healthy patients that are going to be diagnosed with malignant disease during a follow-up period of 3 years.^1^ This latter observation was statistically independent from the association of ACBP/DBI plasma concentrations with age and overweight/obesity,^1^ suggesting that ACBP/DBI is a new risk factor for the future development of cancer.

In mouse experiments, knockout of the *Dbi* gene or antibody-mediated neutralization of circulating ACBP/DBI reduced the propensity to develop rapidly progressive, carcinogen-induced mammary carcinoma and non-small cell lung cancer (NSCLC).^1^ Moreover, the treatment of established fibrosarcoma and NSCLC with chemoimmunotherapy, a combination of the immunogenic cell death inducer oxaliplatin and programmed cell death-1 (PD-1) blockade with a suitable monoclonal antibody (mAb), could be improved by simultaneous neutralization of ACBP/DBI.^1^ Thus, ACBP/DBI may be considered as an immunosuppressive molecule (or ‘immune checkpoint’) the neutralization of which improves immunosurveillance leading to ‘immune checkpoint inhibition’.^3^ Mechanistically, ACBP/DBI has been described as an endogenous inhibitor of autophagy (or ‘autophagy checkpoint’), and mAb-mediated neutralization of extracellular ACBP/DBI (‘autophagy checkpoint inhibition’) is indeed inducing autophagy.^8,9^ Thus, ACBP/DBI mAbs join a long list of pharmacological autophagy enhancers that improve anticancer immunosurveillance.^10–14^

ACBP/DBI is a phylogenetically ancient small protein (87 amino acids) that acts on the gamma-amino butyric acid (GABA) receptor type A (GABA_A_R) and more specifically on one specific GABA_A_R subunit dubbed gamma-2 (gene symbol: *GABRG2*). Indeed, a point mutation in GABRG2 (F77I) is sufficient to abolish the binding of ACBP/DBI to GABRG2 and hence to avoid the metabolic effects of ACBP/DBI.^15–18^ The very same mutation also abrogates the effects of benzodiazepines such as diazepam on GABA_A_R.^19^ This is in line with the long-standing observation that ACBP/DBI can displace diazepam from GABA_A_R,^20,21^ an observation that led to the classification as ACBP/DBI as ‘endozepine’ (for endogenous benzodiazepine).^22,23^ Accordingly, ACBP/DBI and benzodiazepine share similar effects on GABA_A_R as positive allosteric modulators.^24,25^

Driven by these considerations, we wondered whether pharmacological treatment with benzodiazepines might enfeeble immunosurveillance, in particular in the context of inhibition of the endozepine ACBP/DBI. To respond to this question, we attempted to interfere with the immunotherapy-enhancing effect of anti-ACBP/DBI mAb in mouse experiments. Moreover, we analyzed the clinical responses of NSCLC patients to immunotherapy with PD-1 or PD ligand 1 (PD-L1) blocking antibodies for which the therapeutic use of benzodiazepines or other psychotropic drugs was prospectively recorded.

## Materials and methods

### Mouse experimentation

Animal care. Animals were handled following the guidelines provided by the Federation of European Laboratory Animal Science Associations (FELASA) and experimental setups were approved by the local ethical committee (project #24410). All mice were provided with *ad libitum* food supply, collective housing in a light- and temperature-controlled environment with 12-h day/night cycles, and were allowed to rest for one acclimatation week prior to experimentation.

MCA205 tumor challenge. Female C57Bl/6J mice, aged 8-to-10 weeks, were put under light isoflurane anesthesia and injected subcutaneously in the right flank with 3.0 × 10^5^ MCA205 cells. Tumor sizes were measured with an electronic caliper, and mice were assigned to experimental groups with the RandoMice software^26^ to equalize initial tumor burdens at treatment onset (day 8).

**Figure 1.**
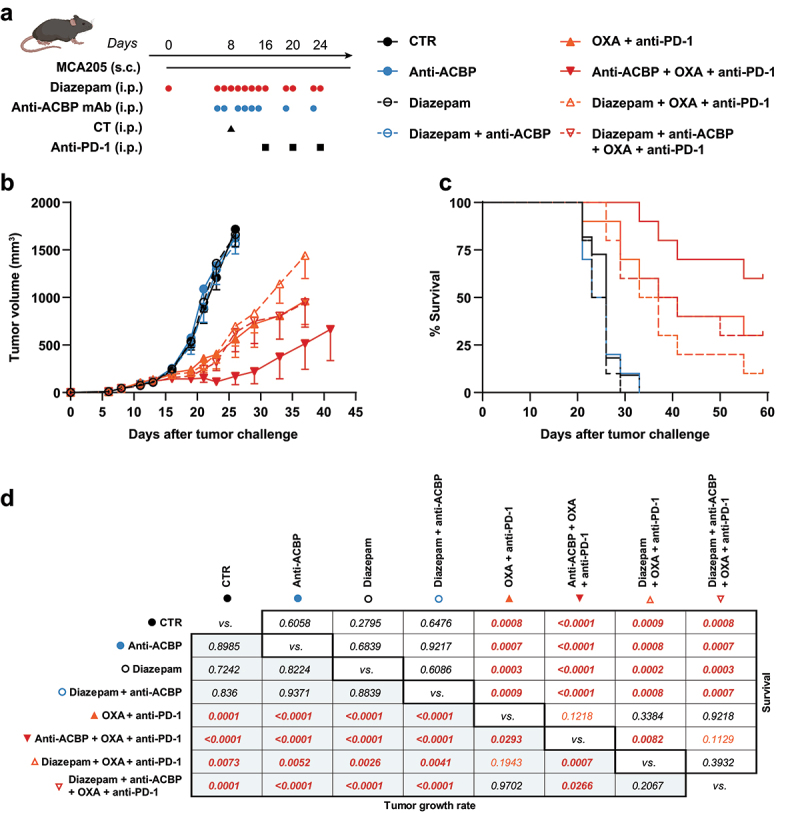
Negative effects of diazepam on immunochemotherapy in mice. Following the schedule of cancer cell inoculation and drug administration to C57BL/6J mice depicted in the scheme (a), the effect of various treatments regimens on the growth of MCA205 fibrosarcomas was assessed. Results are shown as mean tumor size ± standard error of the mean (b) and Kaplan-Meyer plots of the animal survival (c). P-values corresponding to the pairwise comparisons among treatment groups are displayed in a matrix (d). Tumor growth rates were compared with the TumGrowth tool (https://kroemerlab.Shinyapps.io/TumGrowth) by linear mixed effect modeling. Survival differences were tested using log-rank (mantel-cox) test. ACBP: acyl coA binding protein (ACBP/DBI); CT: chemotherapy; PD-1: programmed cell death protein 1.

Drug administration. Treatments were administered following the schedule presented in Figure 1a, with the systematic use of vehicle or isotype controls for all untreated groups. Diazepam (Atnahs, Valium® 1% drinkable solution) or the matching vehicle (40% EtOH, 500 mg/mL propylene glycol [PG, Sigma, #294004] in ddH2O) was diluted to 0.8 mg/mL in a 1:1 PG:ddH_2_O mix and injected intraperitoneally (i.p.) at a dose of 4 mg/kg. This dose corresponds to the clinically relevant dose of 20 mg per day for a 60 kg human adult, following the thumb rule that per-weight doses in mice have to be divided by 12.3 (based on body surface area) to yield per-weight doses in humans.^27^ The custom-made ACBP/DBI-neutralizing monoclonal antibody^1,8^ or the corresponding isotype control (mouse IgG2a, BioXCell, #BE0085) was diluted in PBS and injected i.p. at 5 mg/kg. Oxaliplatin (Sigma, #Y0000271) was dissolved to a concentration of 1 mg/mL in ddH_2_O, sterile-filtered and injected i.p. at 10 mg/kg. PBS was used as the corresponding vehicle control. Anti-PD-1 (BioXCell, #BE0273) or its isotype control (rat IgG2a, BioXCell #BE0089) were diluted in PBS and administered i.p. at a dose of 200 µg per mouse.

### Lung cancer comedications

Clinical data collection was performed under the study ONCOBIOTICS (Sponsor Protocol N: CSET 2017/2619, ID-RCB N: 2017-A02010-53) according to the ethical guidelines and approval of the local ethical committee (Comité Consultatif de Protection des Personnes dans la Recherche Biomédicale (CCPPRB) of the Kremlin Bicêtre Hospital). ONCOBIOTICS is a multicentric prospective observational study recruiting cancer patients with advanced NSCLC treated with anti-PD-(L)1 therapy in France since 2017, patients included in this study were enrolled between October 2019 and November 2023 and their comedications were recorded prospectively. Plasma samples were also obtained through the CRCHUM lung cancer biobank (Ethics number #17.035, Montreal, Canada). Adult patients who signed consent for the CRCHUM biobank, with NSCLC amenable to anti-PD-1 alone or anti-PD-1 in combination with chemotherapy, and without any actionable mutations (EGFR, ALK, ROS) were prospectively included. At each participating center, baseline characteristics of the patients were recorded, including a detailed list of concurrent medications taken in the 2 months before starting immune checkpoint inhibitor treatment, as well as the date of the last follow-up, were entered into an electronic case report form. Patients were stratified based on the concurrent use of GABA_A_R-targeted drugs (benzodiazepines, BZD: adinazolam, alprazolam, climazolam, clobazam, clonazepam, clorazepate, diazepam, estazolam, flunitrazepam, flurazepam, halazepam, lorazepam, loprazolam, lormetazepam, midazolam, nimetazepam, nitrazepam, oxazepam, prazepam, temazepam, triazolam, zolpidem or zopiclone), psychoactive drugs not belonging to this class (PSY not BZD) or none of the latter (others).

### DBI/ACBP ELISA

Plasma concentrations of the endozepine DBI/ACBP were measured by enzyme-linked immunosorbent assay (ELISA) as previously described.^1,17^ In brief, heparin plasmas were diluted 1:50 and incubated for 2 hours on a high-binding plate previously coated with an anti-DBI/ACBP primary antibody (MyBioSource, #MBS768488). Detection was performed by sequential incubation with a biotin-coupled detection antibody against DBI/ACBP (LSBio, #LS‑C299614) for 2 h, followed by avidin-coupled horseradish peroxidase (Biolegend, #405103) for 30 min. Color development was launched by adding the HRP substrate TMB (Thermo Fisher, #34028) for 5–10 min, and absorbance was read at 450 nm after stopping the reaction with 2 M H_2_SO_4_.

### Statistics

Data management and organization were performed in R (v. 4.2.0). Graphical representations were generated with GraphPad Prism (v. 10.3.0). Survival data were plotted as Kaplan–Meier curves and analyzed by log-rank (Mantel-Cox) test, while differences in tumor growth rates were assessed longitudinally by linear mixed effect modeling on the TumGrowth platform (https://kroemerlab.shinyapps.io/TumGrowth/).

## Results

### Effects of diazepam on immunotherapy outcome in mice.

C57BL/6J mice bearing syngeneic orthotopic MCA205 fibrosarcomas (which are derived from 3-methylcholanthrene-induced cancers that originally developed under the skin) can be efficiently treated by chemoimmunotherapy consisting of the combination of systemic intraperitoneal (i.p.) injections of oxaliplatin and a monoclonal antibody (mAb) specific for PD-1, following our standard schedule (Figure 1a). This chemoimmunotherapy regimen can be further improved by neutralization of ACBP/DBI by means of a suitable mAb, as reported.^1^ We optionally combined these treatments of MCA205 fibrosarcomas (in four groups: (i) controls injected with vehicle and an isotype control mAb, (ii) mice injected with anti-ACBP/DBI mAb alone, (iii) mice receiving chemoimmunotherapy and (iv) animals receiving the combination of chemoimmunotherapy and anti-ACBP/DBI mAb) with systemic treatments using the prototypic benzodiazepine diazepam (Figure 1a). Diazepam treatment did not affect the growth of tumors evolving in the control group injected with vehicle and an isotype control nor in mice injected with anti-ACBP/DBI mAb alone (Figure 1b,d, Supplemental Figure S1). Accordingly, diazepam did not alter the survival (until the ethical endpoint) of MCA205 fibrosarcoma-bearing mice treated with vehicle and isotype control or anti-ACBP/DBI mAb alone (Figure 1c,d, Supplemental Figure S1). As reported,^1,28^ chemoimmunotherapy significantly (*p* < 0.0001) reduced the progression of tumors (Figure 1b,d, Fig. S1) and ameliorated animal survival (Figure 1c,d, Fig. S1). Diazepam partially compromised the effects of chemoimmunotherapy on tumor growth, although this effect was not significant (*p* = 0.1943) and barely detectable at the level of animal survival (*p* = 0.3384) (Figure 1b–d, Fig. S1). Of note, diazepam reversed the beneficial effects of anti-ACBP/DBI mAb on the outcome of chemoimmunotherapy (Figure 1b–d, Fig. S1). Of note, diazepam did not affect the bodyweight of the mice, although oxaliplatin did (Fig. S1), meaning that the negative effects of diazepam on chemoimmunotherapy outcome cannot be ascribed to nonspecific toxicity.

In conclusion, diazepam counteracts the capacity of anti-ACBP/DBI mAb to improve chemoimmunotherapeutic effects in mice.

### Effects of benzodiazepines on immunotherapy outcome in patients with non-small cell lung cancer

**Figure 2.**
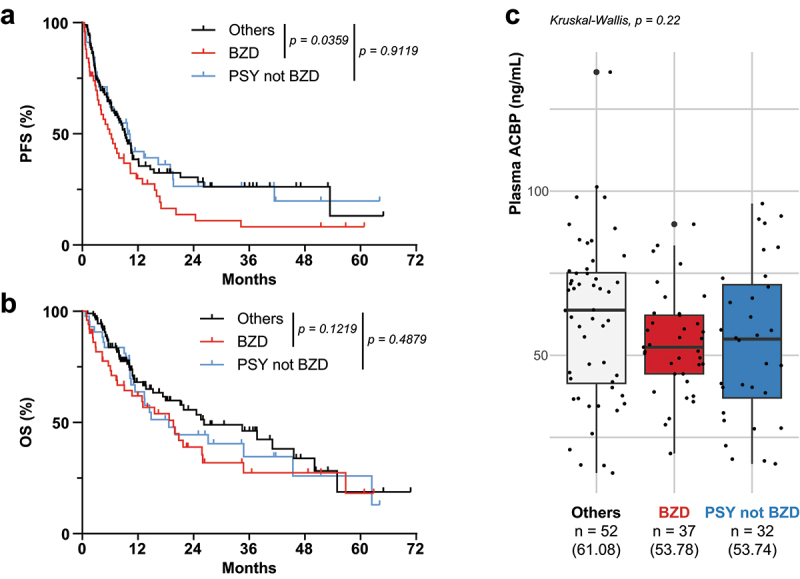
Benzodiazepines and the endozepine ACBP/DBI share a poor prognosis effect in non-small cell lung cancer patients. Kaplan-Meier curves of non-small cell lung cancer (NSCLC) patients included in the ONCOBIOTICS and CRCHUM lung cancer biobank observational studies on comedications demonstrate that the use of benzodiazepines (BZD) during therapy has a negative effect on progression-free survival (PFS, a) and a trend for worsening overall survival (OS, b). These effects were not observed with other psychiatric comedications (PSY not BZD). Circulating levels of ACBP/DBI were quantified by ELISA in the plasma from patients with NSCLC participating to the CRCHUM study (c, *N* = 121 patients with available plasma for ACBP measurements). Survival datasets are displayed as Kaplan-Meier curves and were compared by log-rank Mantel-Cox test, plasma concentrations were compared overall across the three groups by Kruskal-Wallis test.

In the next step, we performed a retrospective analysis of patients with NSCLC. A large spectrum of clinically approved benzodiazepines (such as adinazolam, alprazolam, climazolam, clobazam, clonazepam, clorazepate, diazepam, estazolam, flunitrazepam, flurazepam, halazepam, lorazepam, loprazolam, lormetazepam, midazolam, nimetazepam, nitrazepam, oxazepam, prazepam, temazepam, triazolam, zolpidem or zopiclone) is being used for treating anxiety, dyspnea, panic attacks and sleeping disorders in the general population, as well as in cancer patients.^29,30^ Treatment with benzodiazepines (which act on the same GABA_A_R subunit as ACBP/DBI) has little or no impact on the probability to develop lung cancer.^31–33^ However, when analyzing clinical databases from Gustave Roussy Cancer Center and the University of Montreal Hospital Research Centre (CRCHUM) in which comedications were prospectively recorded (Table S1), we found that treatment of NSCLC cancer patients under (chemo)immunotherapy with benzodiazepines was associated with a significant reduction of progression-free survival (PFS, Figure 2a), as well as with a trend for reduced overall survival (OS, Figure 2b). This negative effect of benzodiazepines was not shared by other psychotropic drugs (Figure 2a,b). It is important to note that patient characteristics were similar across all three comedication groups, including NSCLC stage and Eastern Cooperative Oncology Group (ECOG) performance status (Table S2). Finally, in the subset of CRCHUM patients for whom plasma samples were available, the three groups of patients showed no differences in circulating levels of endogenous ACBP/DBI (Figure 2c).

We conclude that benzodiazepine, but not other psychotropic drugs, may compromise the efficacy of cancer (chemo)immunotherapy.

## Discussion

In this work, we provide evidence that the prototypic benzodiazepine diazepam counteracts the immunostimulatory action of anti-ACBP/DBI neutralization in a mouse model of cancer chemoimmunotherapy. Moreover, we provide a retrospective analysis of a cohort of NSCLC patients undergoing immuno- or chemoimmunotherapy indicating that medication with benzodiazepines but not with other psychotropic agents compromises progression-free survival. Hence, under specific circumstances, benzodiazepines may interfere with (chemo)immunotherapy outcome.

Previous preclinical studies have reported general immunosuppressive effects of benzodiazepines following specific challenges, including a decrease in the humoral and splenic responses to ovalbumin sensitization,^34^ insufficient TNF-α, IL-6 and MCP-1 responses and increased mortality after bacterial infection with *S. pneumoniae*,^35^ as well as a decline in T cell proliferation upon short-term *ex vivo* CD3 stimulation.^36^ The neutralization of the endogenous ACBP/DBI has been shown to decrease inflammation markers in a model of metabolic dysfunction-associated steatohepatitis^9^ and to boost the antitumor T cell response following chemoimmunotherapy.^1^ The combination of these two GABA_A_R-modulating strategies is thus likely to act through immune-dependent mechanisms, which will be worth exploring in the future by precise phenotyping of the resulting humoral and cellular immune responses.

As discussed in the introduction of this work, diazepam (and other benzodiazepines) and ACBP/DBI share a common binding site within the pentameric GABA_A_R, acting on GABRG2 as positive allosteric modulators, hence increasing the effects of GABA on chloride fluxes controlled by GABA_A_R, which is an ionotropic receptor.^15–19,24,25^ While this observation apparently explains the antinomic effects of anti-ACBP/DBI mAb and benzodiazepines, there are also important differences in the mode of action between ACBP/DBI and benzodiazepines.

In the first place, both extracellular ACBP/DBI and benzodiazepines act on additional receptors. Thus, ACBP/DBI and the neuropeptides derived from this protein, in particular octadecaneuropeptide (ODN), can act on a yet-to-be-characterized metabotropic receptor, which is a G protein coupled receptor.^37–39^ Moreover, benzodiazepines can bind to the so-called peripheral benzodiazepine receptor (PBR), which is identical to the 18 kDa translocator protein (TSPO). Ligands of PBR/TSPO are used for TSPO positron emission tomography (PET) imaging of neuroinflammation.^40^ PBR/TSPO ligands, such as RO5–4864, PK11195 and diazepam, have also been used for the experimental induction of apoptosis in cancer cells.^41^ However, if diazepam has negative effects on the outcome of cancer therapy *in vivo*, it appears unlikely that this effect would be mediated by TSPO, which is apparently associated with anticancer effects. Nevertheless, given the diversity of benzodiazepines used in clinics and their potentially different affinities for their respective ligands,^42^ future studies should compare the effects of diazepam with those of other molecules in the same class.

In the second place, antibodies targeting ACBP/DBI and benzodiazepines reach different organs. While antibodies do not penetrate the blood-brain barrier and hence only neutralize the peripheral (extra-central nervous system) pool of ACBP/DBI, clinically used benzodiazepines all affect GABA_A_R in the brain to mediate their anxiolytic and sedative effects.^43,44^ ACBP/DBI induces antinomic effects when injected into the brain (where it exerts anorexigenic effects), compared to the periphery (where it has marked orexigenic effects), supporting the idea that the site of action has a major impact on the behavioral and metabolic effects of ACBP/DBI.^17,29,37–39,45,46^ Of note, some benzodiazepines such as olanzapine induce weight gain as a major side effect,^47,48^ suggesting (but not proving) that they act on GABA_A_R outside of the CNS. Since olanzapine is massively used for the treatment of cancer-anorexia-cachexia syndrome,^49^ as well as for the suppression of chemotherapy-induced nausea and vomiting,^50^ it will be important to understand its potential immunosuppressive side effects.

In summary, our present study suggests that benzodiazepines compromise the outcome of chemoimmunotherapy in mouse models and in NSCLC patients. It will be important to extend these observations to prospective NSCLC cohorts and to investigate the impact of benzodiazepines on immunotherapy in other frequent cancer types. Since benzodiazepines are heterogeneous in their metabolic side effects, it will be interesting to understand whether some benzodiazepines compromise therapy-induced immunosurveillance, while others do not. Hence, further systematic epidemiological studies should determine the precise impact of each drug falling into this category. This applies to benzodiazepines as well as to other selective GABA_A_R modulators.^51^

## Supplementary Material

Table S1_revised.xlsx

Figure S1_revised.eps

Table S2.xlsx

## Data Availability

Detailed patient data was not made available for privacy reasons. The rest of the data that support the findings of this study are available from the corresponding author, GK, upon reasonable request.
